# A Comparative Randomised Controlled Trial of the Effects of Brain Wave Vibration Training, Iyengar Yoga, and Mindfulness on Mood, Well-Being, and Salivary Cortisol

**DOI:** 10.1155/2012/234713

**Published:** 2011-12-15

**Authors:** Deborah Bowden, Claire Gaudry, Seung Chan An, John Gruzelier

**Affiliations:** ^1^Psychology Department, Goldsmiths, University of London, ITC Building, New Cross, London SE14 6NW, UK; ^2^Korea Institute of Brain Science, Caroline Tower, 613-5, Sinsa-dong, Gangnam-Gu, Seoul 135-894, Republic of Korea

## Abstract

This randomised trial compared the effects of Brain Wave Vibration (BWV) training, which involves rhythmic yoga-like meditative exercises, with Iyengar yoga and Mindfulness. Iyengar provided a contrast for the physical components and mindfulness for the “mental” components of BWV. 35 healthy adults completed 10 75-minute classes of BWV, Iyengar, or Mindfulness over five weeks. Participants were assessed at pre- and postintervention for mood, sleep, mindfulness, absorption, health, memory, and salivary cortisol. Better overall mood and vitality followed both BWV and Iyengar training, while the BWV group alone had improved depression and sleep latency. Mindfulness produced a comparatively greater increase in absorption. All interventions improved stress and mindfulness, while no changes occurred in health, memory, or salivary cortisol. In conclusion, increased well-being followed training in all three practices, increased absorption was specific to Mindfulness, while BWV was unique in its benefits to depression and sleep latency, warranting further research.

## 1. Introduction

The presented randomised controlled trial (RCT) with university students compared the effects of Brain Wave Vibration (BWV), Iyengar Yoga, and Mindfulness training on the mood, well-being, and immune function of healthy participants. BWV is an eclectic form of yoga and meditation, involving rhythmic movements of the head, neck, and body. It aims inter alia to tone up the brain's arousal systems including the brain-stem reticular activating system which is a core sleep-energy centre and part of the subcortical-cortical arousal axis controlling energy distribution in the brain and body which was discovered in the 1950s and led to some of the perennial arousal theories in Psychophysiology [1–3]. It is a moving meditation designed to relax mind and body and release negative as well as positive emotions and to induce an increased awareness of the movement of energy [3]. BWV may be performed sitting or standing; the practitioner first gently shakes their head back and forth from the left to right, then follows their own natural rhythm, and focuses on physical sensations and vibrations from the neck and head, which may spread to all parts of the body. BWV is believed to be most beneficial when practiced as part of a holistic fitness class in combination with other related yoga-style exercises, known as “Body and Brain Holistic Fitness Training” or “Dahn yoga”. These mind-body training exercises form part of the Health Smile Peace (HSP) movement, which was developed in South Korea by Il chi Lee in the 1980s, and are designed to optimize body and brain health and rebalance the energy systems of the body [3]. The evidence for the efficacy of BWV training is currently mostly anecdotal, although Jung et al. [4] conducted a trial where participants who had regularly trained in BWV were compared with healthy control participants. They reported an increase in positive affect and plasma DA levels together with a reduction in stress following BWV training, which warrants further research.

In the present study participants underwent a course of BWV training classes, which consisted of a series of Body and Brain Holistic Fitness exercises including BWV. As a comparator for some of the physical components of BWV training, a group of participants also took part in Iyengar Yoga classes. Iyengar yoga practice emphasises standing poses with the aim of building strength, concentration and meditation, and relieving stress. The physical poses aim to increase vitality and improve coordination and balance [5].

A further group of participants underwent a course of Mindfulness classes, intended as a comparator for the “mental” components of BWV. Mindfulness practice is more cognitive, aiming for a present-centered awareness which is nonlaborative and nonjudgmental. Its central component involves self-regulation of attention to control concentration. It also focuses on stress reduction and positive state elevation [6].

The efficacy of the interventions was evaluated pre- and postintervention by scales that were selected to measure behavioral, emotional, and cognitive arousal, negative affect, and mental concentration. In the case of the Depression, Anxiety, and Stress Scale (DASS) [7], this was found apposite in disclosing changes in mood following Reiki in similar university samples to the one recruited here [8, 9]. It was anticipated that improvements in stress reduction would be found in all three groups following the intervention. It was also hypothesized that the BWV group would experience a substantive increase in their vitality levels, as is commonly reported following BWV training [3] and following the theoretical involvement of the brain-stem arousal system described further in the Discussion.

## 2. Materials and Methods

### 2.1. Participants

Following approval by the college Ethics Committee, 45 healthy participants were recruited to take part in the study over a period of four months, through college notice board advertisements. However, only 33 (18–50 years; 21 females, 12 males) completed the study due to 12 dropouts (3 BWV; 6 Iyengar; 2 Mindfulness). Participants were awarded *£*20 on completion of the study.

### 2.2. Design

To assess mood, participants completed the DASS21 [7] at recruitment before they were randomized to the three groups. Then, to prevent the mood scores of the three primary groups, BWV, Iyengar, and Mindfulness, differing at baseline (e.g., [8]), each of the three groups was subdivided into participants with high total DASS scores with scores above 40/126 (High-Mood) and low total DASS scores with scores below 41/126 (Low-Mood). The High- and Low-Mood subgroups were not considered separately in the statistical analyses presented in the Results due to the small number of participants in a subgroup.

As each pair of High-Mood or Low-Mood participants was recruited, they were randomly allocated to the three groups using a computerised random number generator that created a number between 1 and 3, with an equal probability of generating any number. If “1” was generated, the participant was allocated to the BWV group, if “2” was generated, the participant was assigned to the Iyengar group, and if “3” was generated, the participant became a Mindfulness participant. This method ensured that there were equal numbers of High-Mood and Low-Mood participants in each of the three groups, so that the mood scores of the groups would not significantly differ at baseline. When participants dropped out, new recruits continued to be randomly assigned to the three groups by the method described, until the target sample of 12 participants in each group was reached. However, three of the 12 Iyengar participants droppedout at a stage when it was too late to replace them. The experimenter who conducted the randomisation had not met the participants and was naive to the scores of their preassessment measures.

Turning to statistical power, it was calculated using G-Power that the number of participants in each of the groups was insufficient for a significant difference to be observed between three independent samples of equal size. Predicting that there would be an effect size of 1, with an error probability of 0.05 and an allocation ratio of 1, the necessary sample size was calculated to be 15 in each group. Therefore the 12 participants in the BWV and Mindfulness groups and the 9 participants in the Iyengar group were underpowered for an effect of even the high predicted magnitude to be detected. While it would have been preferable to employ a larger sample size to increase statistical power, this was regretfully not possible.

### 2.3. Procedure

Prior to the intervention, participants attended a 60-minute preassessment where the measures detailed in Section 2.4 were administered. An identical assessment was completed following the intervention.

**Timeline of a 60-Minute Assessment**

1. *0 minutes: questionnaire completion (40–45 minutes). *Participants first completed a computerised questionnaire battery consisting of an Excel file containing the questionnaires listed in Section 2.4.
2. *10 minutes: 1st salivary cortisol sample collection (2 minutes).* The questionnaire completion was interrupted by the collection of a salvia sample to assess salivary cortisol.
3. *40 minutes: 2nd salivary cortisol sample collection (2 minutes).*
4. *50 minutes: computerised memory test (The Dual Back *[10]*) (5–10 Minutes). *Finally, participants completed two practice runs of a memory test before the test at preassessment and one practice run at postassessment, in order to familiarise themselves with the procedure.

During the intervention participants attended two 75-minute sessions of BWV, Iyengar, or Mindfulness per week for five consecutive weeks until a total of 10 sessions had been completed. The participants completed the intervention in two cohorts—the first beginning in early January 2010 and the second beginning in mid-February, where in each cohort the participants from each group attended their 10 intervention sessions in classes together, where there were roughly six participants per class in each cohort. Participants were also asked to practice class exercises at home for 10 minutes each day and to record their diligence in a diary. 

#### 2.3.1. Brain Wave Vibration Procedure

The BWV training classes were taught by a trained Body and Brain Holistic Fitness Training instructor and consisted of a series of yoga-like exercises which typically comprise a 75-minute BWV Body and Brain Holistic Fitness class (the BWV itself was just one exercise practiced in the class). The BWV class music usually played at the HSP Centers was played during the classes.

**Timeline of a 75-Minute BWV Class**

1. *0 minutes: Warm Up (15 minutes)*. The classes began with a patting exercise intended to release stagnant energy, stimulate meridians, open acupressure points, and increase energy/blood circulation and awareness of the body.
2. *15 minutes: stretching and rhythm (15 minutes)*. These exercises were designed to open the meridian system and regenerate healthy energy circulation and facilitate improved communication between the body and brain.
3. *30 minutes: breathing postures (15 minutes). *The participants were guided in four breathing postures believed to accumulate energy in the “energy core” of the body, the *Dahn Jon, *since a strong energy core is thought to increase concentration and boost immunity and health [3].
4. *45 minutes: brain wave vibration (10 minutes). *The participants then practiced BWV, which involves rhythmic movements of the neck and head and is believed to create a powerful, peaceful, and positive brain. For the first five classes, participants practiced BWV whilst sitting cross-legged; for the final five classes participants practiced BWV whilst standing, letting the vibrations spread through their whole body in time to the rhythm of the music.
5. *50 minutes: ji-gam (5 minutes)*. Participants practiced an energy meditation designed to quiet the mind, increase focus, lower the frequency of the participant's brain waves, and create peace of mind.
6. *60 minutes: warm down (13 minutes)*. The classes were concluded with stretches and balancing exercises.
7. *73 minutes: sharing (2 minutes)*. Participants sat in a circle at the end of class and shared their experiences.

Participants were requested to perform daily body tapping and BWV exercises, which took a total of 10 minutes. 

#### 2.3.2. Iyengar Yoga Procedure

The Iyengar Yoga sessions were led by a teacher trained to Junior Intermediate level II. The classes involved stretching and breathing exercises and postures [5], performed on yoga mats with the aid of foam bricks and a chair for certain exercises. The class was concluded by a lying down exercise where participants closed their eyes and relaxed. No timeline is included since the specific exercises varied from class to class. Participants were asked to practice their preferred exercises at home for 10 minutes a day.

#### 2.3.3. Mindfulness Procedure

The mindfulness classes were taught by a trained teacher and were designed to teach the participants skills to enable them to become more mindful, so that they may be present in the moment without judging that moment in any way. Mindfulness has many purported benefits, both in enhancing one's life and in dealing with difficult experiences and conditions such as stress, anxiety, and depression [11]. The classes involved various mindfulness exercises, such as sitting and body-scan meditations and group and pair work where participants shared their experiences and enquired into those of others. The classes also involved poetry and exercises to clarify intentions and promote self-understanding. As with the Iyengar yoga classes, the mindfulness class exercises varied from class to class, and thus no timeline is included. Participants were given a 10-minute sitting meditation to practice at home six days a week, and they were also asked to keep a diary to record events.

### 2.4. Assessment Measures

#### 2.4.1. The Depression, Anxiety, and Stress Scale (DASS) [7]

The DASS21 Mood questionnaire is designed to measure negative emotional states of depression, anxiety, and stress. Participants answer from 0 (Not at all) to 3 (Most of the time) on a series of 21 statements.

#### 2.4.2. The Pittsburgh Quality of Sleep Index (PSQI) [12]

The PQSI is a multi-item questionnaire that was used to assess several sleep components over the previous month including sleep disturbances, medication use, tiredness, and apathy. The postassessment version of the scale assessed sleep over the previous week in order for any effects of the intervention to manifest.

#### 2.4.3. The Six-Item Subjective Vitality Scale (SVS) [13]

The Individual Difference Level Version of the SVS was used to measure vitality. The scale asks the respondents to indicate the degree to which the six positively worded statements are true for them in general in their lives. Each item is rated on a 6-point scale (1: not at all true; 2: not true; 3: almost not true; 4: almost true; 5: true; 6: very true). The total score ranges from 6 to 36 with a higher score indicating a better condition.

#### 2.4.4. The Mindfulness Attention Awareness Scale (MAAS) [14]

The MAAS consists of 15 items relating to dispositional mindfulness. The items consist of statements, for example, “I snack without being aware of what I'm eating”, where respondents rate the extent to which each statement is true for them on a scale of 1 (corresponding to “Almost always”) to 7 (corresponding to “Almost always”). The scale is scored by computing a mean of the 15 items.

#### 2.4.5. The Visual Analogue Scale of BWV Benefits (VAS)

A visual analogue scale was developed for the study designed to measure benefits commonly reported to occur in those who practice BWV, although as it was not possible to validate the scale the results obtained must be interpreted with caution. The scale consists of 20 statements: “I have had high vitality levels”; “I have felt tense”; “I have had headaches”; “I have felt confident”; “I have had aches and pains”; “I have felt calm”; “I have found it easy to focus”; “I have slept well”; “My body has felt flexible”; “I have felt aware of my emotions”; “My digestion has been good”; “I have experienced self-doubt and insecurities”; “I have had good circulation”; “I have had a positive outlook on life”; “My head has felt clear and not foggy”; “I have felt grounded”; “I have found it easy to cope with life's stresses”; “I have felt aware of physical sensations in my body”; “I have felt in control of my emotions”; “I have enjoyed good interpersonal relationships”. Participants assign a percentage score to each statement to indicate the extent to which the statement is true for them. Each statement was considered separately in the analysis presented in Section 3.

#### 2.4.6. The Tellegen Absorption Scale (TAS) [15]

The TAS is a 34-item scale designed to measure a person's openness to absorbing and self-altering experiences. Respondents rate the extent to which statements which would describe a person with high absorption apply to themselves—for example, “the crackle and flames of a wood fire stimulate my imagination”. Participants answer each item on a scale of 1 to 7, where 1 corresponds to “never”, 4 corresponds to “sometimes”, and 7 corresponds to “always”.

#### 2.4.7. The Illness Symptoms Questionnaire [8, 9]

The ISQ is a simple 20-item questionnaire that assesses the presence of illness symptoms, such as fever and runny nose, where participants state how many days in the previous two weeks each symptom had been experienced. The absence of a symptom corresponded to a score of 0, a symptom present for 1-2 days a score of 1, a symptom present for 3-4 days a score of 2, a symptom experienced for 4–6 days a score of 3, and a symptom present for 7–14 days a score of 4. The severity score of each symptom was then summed to form a Total Illness score for each participant.

#### 2.4.8. The Dual-Back Task (2-Back) [10]

The 2-Back is a task which assesses working memory, where the participant is presented simultaneously with two different sequences of stimuli and they must indicate when the current stimulus from each sequence matches the stimulus from two steps earlier in that sequence. Here participants were presented with a sequence of images on a computer screen of a ball positioned at various positions on a grid, in addition to an auditory string of alphabetical letters. Participants attempted to recall the position of the ball on the grid and also the auditory letter that was simultaneously presented. Participants were scored on their accuracy.

#### 2.4.9. Salivary Cortisol Assessment

Cortisol is understood to be a stress hormone and also to be involved in immunological defence, and salivary cortisol has been demonstrated to provide an accurate index of blood cortisol [16].

In order to assess cortisol levels (nmol/L), saliva samples were collected from participants, who were given Sarstedt salivettes containing a cotton wool swab, which they chewed for 1-2 minutes. The samples were stored in a −80°C fridge until the end of the study, when they were sent to a laboratory to be analysed using ELISA assay kits. To maximise the reliability of cortisol readings, two saliva samples were collected at both pre- and postassessment, and the mean of the two readings is taken. Additionally, saliva was collected between 11 am and 3 pm, when cortisol levels are relatively stable, with preassessment collection times matched to postassessment collection times. Participants were requested to abstain from caffeine, alcohol, food, and exercise 2 hours prior to assessment to minimise the potential influence of these variables on cortisol readings [17]. Participants completed a short check-list reporting compliance with restrictions, which also assessed their sleep time and level.

### 2.5. Statistics

The efficacy of the trial was primarily assessed using Mixed ANOVA to compare the preintervention (Pre) and postintervention (Post) scores of each of the measures completed by participants (the DASS, the PSQI, the SVS, the MAAS, the VAS, the TAS, the ISQ, the 2-Back, and salivary cortisol). The between-subjects factor was Group (BWV, Iyengar, and Mindfulness) and the within-subjects factor was Session (Pre and Post). Planned contrasts within group changes were done with paired *t*-tests.

## 3. Results

The diaries completed by participants indicated that they had performed their home practice a mean of 4.04 (SD: 1.17) days per week, a one-way ANOVA finding no statistical differences between the groups (*F* = 1.651, ns).

There were no baseline differences in the scores of the groups for the DASS, the PSQI, the SVS, the MAAS, the TAS, the VAS, the ISQ, or salivary cortisol, as disclosed by one-way ANOVAs (*F* ≤ 2.447, *P* ≥ 0.104). It was thus considered reasonable to compare the mean changes of the groups for each of these measures. There was a tendency though for the baseline scores of the 2-Back to statistically differ for the different groups (*t* = 3.095, *P* = 0.06), where a Post hoc Tukey test found a trend for the Iyengar participants to have significantly lower scores than the Mindfulness participants (Mean Difference: −17.444, *P* = 0.072), although the differences between the Iyengar and BWV groups (Mean Difference: −15.944, ns) and the BWV and Mindfulness groups (Mean Difference: −1.5, ns) were nonsignificant.

### 3.1. The Depression, Anxiety, and Stress Scale [7]

Considering first the mood questionnaire used to select participants, the means and standard deviations for the sum total of DASS items, Total DASS, and for the subscales Depression, Anxiety, and Stress, are shown in Table 1. Two Mindfulness participants were excluded from the DASS analysis—one participant whom had a Pre Anxiety score that was 3.2 SDs above the sample mean, and one participant whom had a Pre Depression score 2.96 SDs above the sample mean.

**Figure 1:**
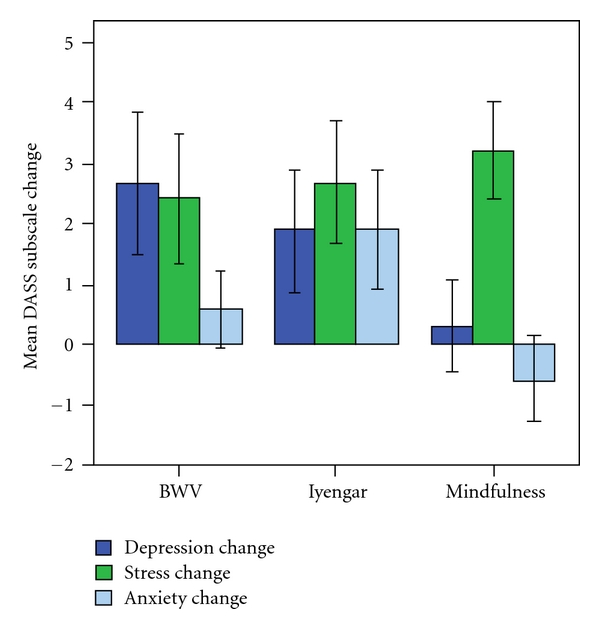
The mean (SE) change in the DASS subscales for the BWV, Iyengar, and Mindfulness groups, where an increase corresponds to an improvement.

Mood improved overall the whole sample, as disclosed by a mixed ANOVA which found a significant main effect of Session for the sum total of DASS items, Total DASS (*F*(1, 28) = 19.617, *P* < 0.001). The mean (SD) changes in the Total group scores were as follows: BWV decrease = 5.67 (6.21); Iyengar decrease = 6.44 (7.07); Mindfulness decrease =  3.5 (6.39)). Separate ANOVAs on the subscales indicated that the overall improvement was largely due to reductions in both Stress (*F*(1, 28) = 23.596, *P* < 0.001) and Depression (*F*(1, 28) = 7.148, *P* = 0.012), for reductions in Anxiety were not significant (*F*(1, 28) = 2.223, ns). Subscale results are shown in Figure 1.

 While there were no Group x Session interactions, planned contrasts within groups with paired *t*-tests found for the BWV group a decrease in Total scores (*t*(1, 11) = 3.159, *P* = 0.009) and in the subscales of Depression (*t*(1, 11) = 2.24, *P* = 0.047) and Stress (*t*(1, 11) = 2.339, *P* = 0.039), although the change in Anxiety was nonsignficant (*t*(1, 11) = 0.904, ns). In the Iyengar group there was a significant decrease in the Total score (*t*(1, 8) = 2.733, *P* = 0.026) and in Stress (*t*(1, 8) = 2.596, *P* = 0.032) but not in the other subscales (Depression: *t*(1, 8) = 1.828, ns; Anxiety: *t*(1, 8) = 1.931, ns), while in the Mindfulness group there was a reduction in Stress (*t*(1, 9) = 3.932, *P* = 0.003), and no other effects (Total DASS: *t*(1, 9) = 1.759, ns; Depression: *t*(1, 9) = 0.402, ns; Anxiety: *t*(1, 9) = −0.874, ns).

In summary, there were improvements in Total scores following BWV and Iyengar, improvements in Stress for all groups, for Depression with BWV, while none of the groups improved in Anxiety.

### 3.2. The Pittsburg Sleep Quality Index [12]

Table 7 shows the mean Pre and Post scores for the sum total of PSQI items, Global Sleep, and for each of the PSQI subscales. 

The sample as a whole showed no significant change in Global Sleep (Session: *F*(1, 30) = 1.84, ns). Considering the groups separately with paired *t*-tests also disclosed no significant changes (BWV:  *t*(1, 11) = 1.503, ns; Iyengar: *t*(1, 8) = 0.223, ns; Mindfulness: *t*(1, 11) = 0.639, ns).

**Figure 2:**
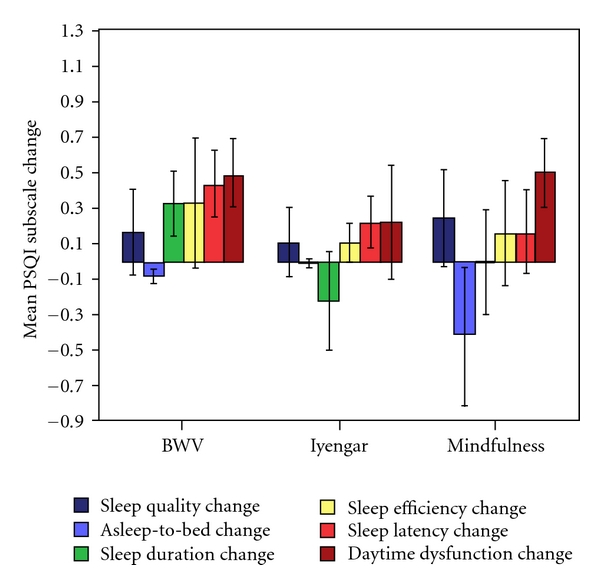
The mean (SE) group changes in the PSQI subscales, where a positive change indicates an improvement.

Significant changes were found for certain PSQI subscales however. The mean changes of the three groups for each of the subscales are shown in Figure 2, except for Sleep Disturbance, as no participant showed any change in this subscale. Sleep Latency was found to significantly decrease in the whole sample (Session: *F*(1, 30) = 5.315, *P* = 0.028). Paired *t*-tests found a significant decrease in the BWV group (*t*(1, 11) = 2.321, *P* = 0.04), while neither of the other groups showed significant changes (Iyengar: *t*(1, 8) = 1.512, ns; Mindfulness: *t*(1, 11) = 0.692, ns).

There was also a marked improvement in the subscale Daytime Dysfunction in the whole sample, where the main effect of Session was highly significant (*F*(1, 30) = 9.19, *P* = 0.005). Both the BWV and Mindfulness groups were found by paired *t*-tests to have significantly lower mean scores following the intervention (BWV: *t*(1, 11) = 2.569, *P* = 0.026; Mindfulness: *t*(1, 11) = 2.569, *P* = 0.026), although there was no notable change in the Iyengar group (*t*(1, 8) = 0.686, ns). 

No main effects of Session were found for the other PSQI subscales however (Sleep Quality: *F*(1, 30) = 1.446, ns; Asleep-to-bed: *F*(1, 30) = 1.348; ns, Sleep Disturbance: *F*(1, 30) = 0, ns; Sleep Duration: *F*(1, 30) = 0.056, ns; Sleep Efficiency: *F*(1, 30) = 1.292, ns). There were also no Session x Group effects found for the total PSQI score or for any of the subscales (*F*(2, 30) ≤ 1.078, *P* ≥ 0.353).

In summary, the BWV group showed the greater benefit in time to fall asleep and a benefit shared with Mindfulness with residual daytime sleepiness. 

### 3.3. The Subjective Vitality Scale [13]

**Figure 3:**
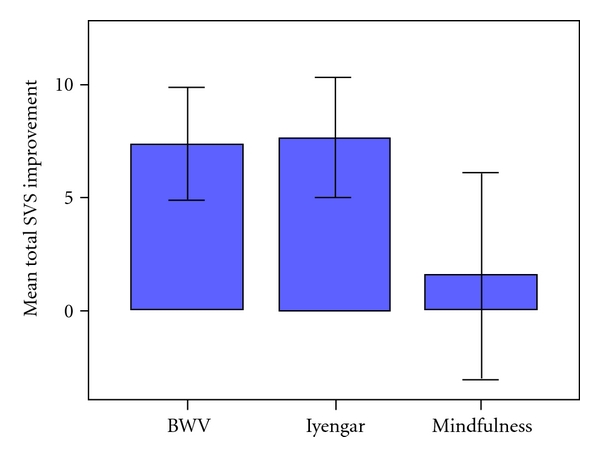
The mean improvement (SE) in the Total SVS scores of the three groups.

The mean Pre and Post scores for the sum total of SVS items, Total SVS, are shown in Table 2. There was an increase in vitality in the sample overall, as can be seen by the change in the Total SVS score in Figure 3 (*F*(1, 30) = 7.208, *P* = 0.012). Paired *t*-tests disclosed marked improvements in the BWV (*t*(1, 11) = −2.931, *P* = 0.014) and Iyengar (*t*(1, 8) = −2.881, *P* = 0.02), but little change in the Mindfulness group (*t*(1, 11) = −0.343, ns).

### 3.4. The Mindfulness Attention Awareness Scale [14]

Table 3 shows the mean Pre and Post scores of the three groups and the sample overall for the mean of MAAS items, Mean MAAS.

**Figure 4:**
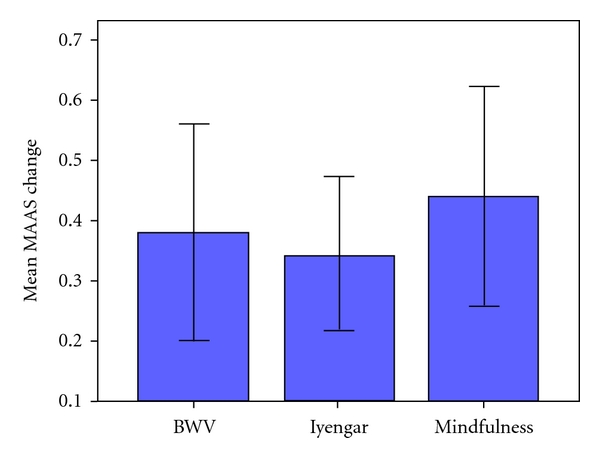
Mean (SE) change in the Mean MAAS scores of the BWV, Iyengar, and Mindfulness groups.

There was an increase in Mindfulness for sample as a whole (*F*(1, 30) = −2.39, *P* = 0.036). Paired *t*-tests indicated significant effects for the Mindfulness (*t*(1, 11) = −2.688, *P* = 0.028) and Iyengar (*t*(1, 8) = −2.688, *P* = 0.028) groups and an effect approaching significance in the BWV group (*t*(1, 11) = −2.082, *P* = 0.062). These increases can be seen in Figure 4. 

 As with the previous scales, though, there were no statistical differences in the comparative changes of the three groups, and the Group x Session interaction was nonsignificant (*F*(2, 30) = 0.075, ns).

### 3.5. The Tellegen Absorption Scale [15]

The mean and standard deviation Pre and Post scores for the sum total of TAS items, Total TAS, are shown in Table 4.

**Figure 5:**
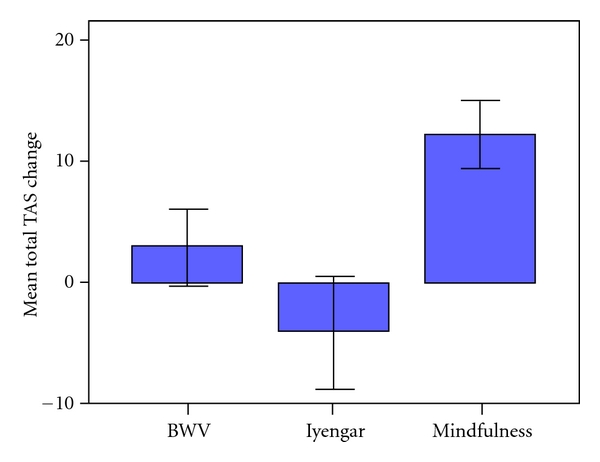
Mean (SE) change in the Total TAS scores of the BWV, Iyengar, and Mindfulness groups.

There was a marginal increase in absorption in the sample overall, marked by a slight tendency towards a main effect of Session (*F*(1, 30) = 3.254, *P* = 0.081). As can be seen in Figure 5 the Mindfulness group accounted for most of the improvement (*t*(1, 11) = −4.322, *P* = 0.001; BWV: *t*(1, 11) = −0.896, ns; Iyengar: *t*(1, 8) = 0.833, ns), supported by a significant Session x Group interaction (*F*(2, 30) = 4.992, *P* = 0.013).

### 3.6. The Visual Analogue Scale of BWV Benefits

The mean and standard deviations of the Pre and Post scores for each of the items of the VAS are shown in Table 8.

In accordance with the increase in vitality seen with the SVS, the VAS also indicated that vitality increased in the whole sample, disclosing a main effect of Session for the item “I have had high vitality levels” (*F*(1, 30) = 6.764, *P* = 0.014). Separate paired *t*-tests indicated that this increase approached significance in both the BWV (*t*(1, 11) = −1.985, *P* = 0.078, mean increase: 15.3% (SD: 26.6%)) and Mindfulness (*t*(1, 11) = −2.018, *P* = 0.069, mean increase: 14.5% (SD: 28.7%)) groups, but not in the Iyengar group (*t*(1, 8) = −0.989, ns, mean increase: 9.4% (SD: 29.6%)).

For the item “I have felt tense” there was tendency towards a reduction overall (Session: *F*(1, 30) = 3.398, *P* = 0.061). There was a substantive decrease in the BWV group (*t*(1, 11) = 3.427, *P* = 0.006, mean decrease: 31.3% (SD: 31.7%)) not seen in the other groups (Iyengar: *t*(1, 8) = −0.106, ns, mean increase: 1.67% (SD: 47.4%); Mindfulness: *t*(1, 11) = −0.701, ns, mean decrease: 5.83% (SD: 44.5%)).

There was a general increase for the item “I have felt calm” (*F*(1, 30) = 4.449, *P* = 0.043), attributable to the Mindfulness group (*t*(1, 11) = −2.204, *P* = 0.05, mean increase: 18.8% (SD: 44.5%)) (BWV: *t*(1, 11) = −1.733, ns, mean increase: 15.3% (SD: 30.7%); Iyengar: *t*(1, 8) = −0.171, ns, mean increase: 2.22% (SD: 38.9%)).

The sample as a whole also tended to feel more grounded following the intervention, as evinced by the item “I have felt more grounded” (*F*(1, 30) = 3.882, *P* = 0.058), but with paired *t*-tests again disclosing a significant effect only for the Mindfulness group (*t*(1, 11) = −2.62, *P* = 0.024, mean increase: 17.9% (SD: 23.7%)) (BWV: *t*(1, 11) = −0.599, ns, mean increase: 7.67% (SD: 44.3%); Iyengar: *t*(1, 8) = −0.947, ns, mean increase: 14.9% (SD: 47.2%)).

Separate Mixed ANOVAs for each of the other VAS items found a significant main effect of Session for the item “I have had good circulation” (*F*(1, 30) = 4.192, *P* = 0.049) and a tendency towards a main effect of Session for the item “My head has felt clear and not foggy” (*F*(1, 30) = 3.659, *P* = 0.065), where for both items an increase was seen in the sample as a whole with no group differences. There were no main effects of Session for the remaining 18 VAS items (*F*(1, 30) ≤ 2.927, *P* ≥ 0.097). In addition, no Group x Session interactions were found for any subscale (*F*(2, 30) ≤ 2.117, *P* ≥ 0.138).

In summary, for the sample as a whole there were improvements in feelings of vitality, tension, calmness, groundedness, circulation, and clear-headedness, with advantages following BWV for vitality and tension, and following Mindfulness for vitality, calmness, and groundedness. 

### 3.7. The Illness Symptoms Questionnaire [8, 9] and the Dual-Back Task [10]

The means and standard deviations for the Pre and Post scores for the sum total of Illness Symptoms Questionnaire items, Total ISQ, and for the Dual Back are shown in Table 5.

The whole sample showed no change in either illness symptoms or working memory, and the main effects of Session for Total Illness and 2-Back were both nonsignificant (Total Illness: *F*(1, 30) = 0.456, ns; 2-Back: *F*(1, 30) = 0.072, ns), nor was there a Session x Group interaction for either of these variables (Total Illness:  *F*(2, 30) = 0.047, ns; 2-Back: *F*(2, 30) = 0.196, ns).

### 3.8. Salivary Cortisol

Table 6 shows the mean and standard deviation Pre and Post Salivary cortisol scores of the three groups and the sample overall. There was a slight increase in salivary cortisol in the whole sample, disclosed by a tendency towards a main effect of Session (*F*(1, 30) = 3.373, *P* = 0.073). However, paired *t*-tests indicated that the increase was not significant for any individual group (BWV: *t*(1, 11) = −1.022, ns; Iyengar: *t*(1, 8) = −1.635, ns; Mindfulness:  *t*(1, 11) = −0.749, ns). The Session x Group interaction was also nonsignificant (*F*(2, 30) = 0.093, ns). 

## 4. Discussion

There were significant improvements for the sample as a whole for many of the outcome measures. However, separate group analyses disclosed that only for the Stress and Mindfulness scales were effects significant for all three interventions, with the majority of improvements found for one or two groups only. There was only one significant interaction between Group and Session which was found for Absorption and this strongly characterized training in Mindfulness. Considering reliable improvements as defined by Group effects with *P* ≤ 0.01, BWV training advantaged the Total DASS mood score (*P* = 0.009), the Subjective Vitality Scale (*P* = 0.014), and the item “I have felt tense” (*P* = 0.006); Mindfulness training, aside from absorption (*P* = 0.006), advantaged Stress (*P* = 0.003); Iyengar yoga did not result in any improvements reaching the *P* ≤ 0.01 level. Considering further group effects, the most improvements were seen following training in Brain Wave Vibration. BWV training was alone in producing significantly better Depression and Sleep Latency. The increase in vitality as evinced by the total SVS score was endorsed by the Visual Analogue Scale item “I have had high vitality levels” which approached significance. Turning to Iyengar training, improvements were found for Total DASS and SVS vitality—improvements found with the BWV intervention, but not with Mindfulness training. The Mindfulness group on the other hand produced improvements in several items that were not seen following other interventions. As noted above, Mindfulness significantly increased absorption, which is in keeping with the findings of Lau et al. [18], who found the Tellegen Absorption Scale to be positively correlated with subscales of the Toronto Mindfulness Scale. Mindfulness training was also the only one to result in significant improvements in the VAS items “I have felt calm” and “I have felt grounded”. In addition, Mindfulness training shared with BWV training reductions in the VAS vitality item and in daytime sleepiness.

The sample as a whole also had a tendency towards an increase in the stress hormone salivary cortisol, despite improvements in both stress and sleep subscales. This relation has been observed following other mind-body interventions, where in one study an increase in plasma cortisol accompanied in the face of exam stress resilience in lymphocyte suppression [19], in another resilience in succumbing to an influenza epidemic [20], and has been correlated with reductions in tiredness [20, 21]. While cortisol may be linked with chronic stress, in the short term it is also associated with preparedness, as with its rise on waking [22].

It is possible that the positive effects disclosed by the trial may have been stronger if the sample size had been larger, for the 12 participants per group were underpowered for a between-group effect of even a very large magnitude to be detected. The benefits of the trial may also have been limited by the general good health of the university students—it is possible that if the trial were conducted with a patient sample, then greater group differences may be observed. A further shortcoming of the trial was that no follow-up data were collected; so it is not known for how long the observed benefits were maintained. Additionally, the participants only trained for the relatively short period of five weeks. It would also be of interest to investigate whether participants who attended a greater number of classes would demonstrate greater improvements, since, for example, benefits following BWV are reported to manifest more strongly after a longer training period [3] and 5 weeks would generally be regarded as a short period to achieve sustained benefits from Ivengar yoga.

Given that the research design, university participant sample, and time in the academic year in the present study were similar to controlled studies of Reiki [8, 9] some comparisons may be made between the trial outcomes. In the first Reiki study [8] there was also a reduction in Stress on the DASS and this was found to be accompanied by an improvement on the Illness Symptom Questionnaire. In the second study [9], where participants were selected on similar mood criteria as in the present study and divided into high- and low-mood groups, while there was no improvement in illness symptoms, there was a reduction in both DASS Anxiety and Stress. These reductions were found to be specific to the high- (negative) mood subgroup following Reiki, and importantly they were sustained at follow-up.

Turning to a number of theoretical issues, considerations have been given to the construction of scalar measures to capture the impact of mindfulness training [11, 18]. Here it is of interest that while all three interventions disclosed improvements on the Mindfulness Attention Awareness Scale [14], it was the Tellegen Absorption Scale [15] that differentiated the meditational effects of Mindfulness training compared with Iyengar yoga and BWV. Indeed Absorption was the only reliable differential characterization of a single intervention in the present trial. Absorption is in keeping with the particular cognitive aspect of mindfulness training when compared with the physical exercise components of both Iyengar yoga and BWV training.

BWV in terms of measured outcome was successful in benefitting both ends of the mood/arousal continuum. This was in keeping with the putative involvement of the brain-stem arousal sleep/wake regulatory systems [1–3]. The brainstem reticular (netlike) formation first arose in the 19th century and from the middle of the 20th century gained prominence following electrophysiological studies of Moruzzi and Magoun [1] as a neuronal system responsible inter alia for the regulation of arousal [2, 23]. In evolutionary terms it is the oldest part of the brain situated at the junction between the spinal cord, cerebellum, and higher limbic and cortical structures and thereby strategically situated through its white and gray matter for roles in the mediation, modulation, and connectivity of vital functions necessary for life including cardiovascular control, and with devastating consequences in the case of even small lesions such as irreversible coma. The historical homogeneity of the reticular formation has given way to functional, anatomical, and biochemical heterogeneity [24]. It receives impulses from the musculoskeletal frame, internal milieu, viscera, and vestibular organs which map the state of the organism and through ascending and descending systems allows regulation of homeostasis in collaboration with thalamocortical and hypothalamic systems. Impulses from all sensory systems underpin its essential role in the control of sleep/wakefulness transitions. Through sensory gating to the cortex fundamental processes for attention such as habituation are controlled. Coincidental with increased alertness and attention there is increased blood flow in the midbrain reticular formation and intra thalamic nuclei [25]. With regard to somatic motor control axons from reticulospinal tracts send signals in order to maintain balance, tone, and posture. It is hypothesised that the rhythmic actions of the BWV protocol as well as the focusing of attention on the actions facilitate such regulatory processes.

While BWV was effective in reducing overarousal as indexed by measures of stress and tension, as was the case with the other two interventions, BWV was alone in having an impact on the low end of the arousal dimension manifested in the reduction in depression. This was also the implication from a cross-sectional investigation of practitioners and controls [4] where positive affect was higher in practitioners and there was a nonsiginificant trend for negative affect to be reduced. BWV was also distinguished by its benefits for shortening the time taken to fall asleep seen in the Sleep Latency score. BWV training also reduced daytime sleepiness, as did Mindfulness training. An improvement in sleep has been a common anecdotal report by BWV practitioners, as has an increase in energy and vitality. Here the latter outcome as measured by the Vitality scale was shared with Iyengar yoga. This is the first controlled trial of this form of mind-body training and the results are sufficiently provocative to warrant further investigation of a unique integration and evolution of meditational and yogic practice.

## 5. Conclusion

In conclusion, substantive benefits to measures of mood and well-being were seen in all three of the groups in this trial, while in no measure was a significant negative change seen in any group, suggesting that all three practices are beneficial. Regarding the BWV group, the substantive improvements in mood, sleep subscales, vitality, and mindfulness, while not significantly greater than the improvements in the other groups, are in accordance with the reported benefits of BWV [3] and with hypothesis. Further randomised controlled trials of the effects of BWV training are warranted to explore the effects found and to isolate which exercises give rise to them, preferably employing larger sample sizes in order to increase statistical power.

## Figures and Tables

**Table 1:** Means (SD)* of total dass, depression, anxiety, and stress**.

	Total DASS		Depression		Anxiety		Stress	
	Pre	Post	Pre	Post	Pre	Post	Pre	Post
BWV	14.9	9.3	5.4	2.8	2.5	1.9	7.0	4.6
	(8.3)*	(8.6)	(4.1)	(3.3)	(1.6)	(2.9)	(4.4)	(3)
Iyengar	18.7	12.2	5.4	3.6	4.3	2.4	8.9	6.2
	(7.6)	(6.5)	(2.7)	(2.7)	(2.6)	(1.6)	(3.7)	(4.1)
Mindfulness	13.6	10.1	2.9	2.6	2.5	3.1	7.6	4.4
	(5.6)	(8.3)	(1.9)	(2.7)	(2.4)	(3.8)	(3.1)	(2.5)
Sample total	15.6	10.4	4.6	2.9	3.0	2.5	7.7	5.0
	(7.4)	(7.8)	(3.3)	(2.9)	(2.3)	(2.9)	(3.8)	(3.2)

*Standard deviations are shown in parentheses. **A lower score indicates better mood.

**Table 2:** Mean (SD)* of Total SVS**.

	Total SVS	
	Pre	Post
BWV	22.2	29.6
	(10.9)*	(7.6)
Iyengar	17.7	25.3
	(6.7)	(6.4)
Mindfulness	22.6	24.2
	(12)	(10)
Sample Total	21.1	26.5
	(10.3)	(8.4)

*Standard deviations are shown in parentheses. **A higher score indicates higher vitality.

**Table 3:** Mean (SD)* of Mean MAAS**.

	Mean MAAS	
	Pre	Post
BWV	3.8	4.1
	(0.7)*	(0.6)
Iyengar	3.7	4.0
	(1.1)	(0.8)
Mindfulness	3.6	4.0
	(0.6)	(0.6)
Sample Total	3.7	4.1
	(0.8)	(0.7)

*Standard deviations are shown in parentheses. **A higher score indicates higher mindfulness.

**Table 4:** Mean (SD)* of Total TAS**.

	Total TAS	
	Pre	Post
BWV	130.3	133.3
	(29.9)*	(32.2)
Iyengar	132.1	128.1
	(33.5)	(33.7)
Mindfulness	130.4	142.7
	(23.9)	(24.9)
Sample Total	130.8	135.3
	(28.0)	(29.8)

*Standard deviations are shown in parentheses. **A higher score indicates higher absorption.

**Table 5:** Mean (SD)* of the Total ISQ and the 2-Back**.

	Total ISQ		2-Back	
	Pre	Post	Pre	Post
BWV	9.2	6.9	53.2	52.2
	(8.5)*	(5.1)	(15.2)	(27.3)
Iyengar	10.7	9.7	37.2	38.6
	(13.8)	(10.2)	(20.9)	(22.8)
Mindfulness	11.3	10.4	54.7	54.3
	(8.6)	(9.7)	(16.2)	(16.4)
Sample Total	10.4	8.9	49.4	49.6
	(9.9)	(8.3)	(18.3)	(22.8)

*Standard deviations are given in parenthesis. **A higher score indicates greater illness symptoms and working memory, respectively.

**Table 6:** Mean (SD)* salivary cortisol.

	Salivary cortisol	
	Pre	Post
BWV	0.23	0.28
	(0.16)*	(0.25)
Iyengar	0.12	0.19
	(0.11)	(0.13)
Mindfulness	0.13	0.17
	(0.06)	(0.17)
Sample Total	0.16	0.21
	(0.12)	(0.19)

*Standard deviations are shown in parenthesis.

**Table 7:** Mean (SD)* of Global Sleep and the PSQI subscales**.

		BWV	Iyengar	Mindfulness	Sample Total
Global Sleep	pre	8.8	7.12	8.45	8.21
		(3.59)*	(1.74)	(3.86)	(3.29)
	post	7.44	7.02	7.78	7.45
		(3.51)	(1.87)	(2.9)	(2.84)
Sleep Quality	pre	2	1.89	2.08	2
		(0.74)	(0.6)	(1.08)	(0.83)
	post	1.83	1.78	1.83	1.82
		(0.72)	(0.44)	(0.72)	(0.64)
Asleep-to-bed	pre	0.8	0.9	0.86	0.85
		(0.18)	(0.09)	(0.11)	(0.14)
	post	0.88	0.9	1.28	1.03
		(0.11)	(0.06)	(1.37)	(0.83)
Sleep Disturbance	pre	1	1.11	1	1.03
		(0.43)	(0.78)	(0.43)	(0.53)
	post	1	1.11	1	1.03
		(0.43)	(0.78)	(0.43)	(0.53)
Sleep Duration	pre	1.17	0.44	0.92	0.88
		(1.19)	(0.73)	(1.08)	(1.05)
	post	0.83	0.67	0.92	0.82
		(1.03)	(0.71)	(1.08)	(0.95)
Sleep Efficiency	pre	0.83	0.22	0.5	0.55
		(1.19)	(0.44)	(1)	(0.97)
	post	0.5	0.11	0.33	0.33
		(0.8)	(0.33)	(0.65)	(0.65)
Sleep Latency	pre	1.75	1.22	1.58	1.55
		(0.87)	(0.44)	(0.79)	(0.75)
	post	1.31	1	1.42	1.26
		(0.67)	(0)	(0.67)	(0.58)
Daytime Dysfunction	pre	1.5	1.33	1.25	1.36
		(0.8)	(0.71)	(0.87)	(0.78)
	post	1	1.11	0.75	0.94
		(0.6)	(0.6)	(0.62)	(0.61)

*Standard deviations are shown in parentheses. **A lower score corresponds to better sleep.

**Table 8:** Mean (SD) of each VAS item (%).

		BWV	Iyengar	Mindfulness	Sample Total
I have had high vitality levels	Pre	52.9	58.9	46.8	52.5
		(17.1)*	(26.7)	(25.7)	(22.9)
	Post	68.2	68.3	64.2	66.8
		(12.2)	(14.8)	(20.4)	(15.9)
I have felt tense	Pre	60	45	48.2	51.7
		(23.6)	(30.6)	(26.1)	(26.5)
	Post	28.7	46.7	38.3	37.1
		(21.9)	(27.5)	(20.5)	(23.5)
I have had headaches	Pre	25.4	22.8	18.3	22.1
		(29.2)	(28.6)	(28.2)	(27.9)
	Post	24.3	11.6	9.2	15.3
		(33.6)	(19.3)	(17.7)	(25.2)
I have felt confident	Pre	52.9	59.8	66.3	59.6
		(23.2)	(18.4)	(21.1)	(21.4)
	Post	64.4	59.4	65.4	63.4
		(19.2)	(28.4)	(17.1)	(20.9)
I have had aches and pains	Pre	31.7	35.6	32.1	32.9
		(32.1)	(27.8)	(31.1)	(29.7)
	Post	24.2	31.7	39.2	31.7
		(28.7)	(25.5)	(35.7)	(30.5)
I have felt calm	Pre	50	51.7	47.1	49.4
		(22.3)	(18.4)	(20.4)	(20)
	Post	65.3	53.9	65.8	62.4
		(20.1)	(26.2)	(13.1)	(19.9)
I have found it easy to focus	Pre	41.3	38.3	48.3	43
		(24.7)	(25.2)	(18.1)	(22.4)
	Post	49	47.1	61.7	53.1
		(26.2)	(27.1)	(19.9)	(24.5)
I have slept well	Pre	59.5	61.7	62.5	61.2
		(27.5)	(32.9)	(18.6)	(25.5)
	Post	68.1	66.1	65.4	66.6
		(32.6)	(20.9)	(29.2)	(27.7)
My body has felt flexible	Pre	47.5	62.2	39.6	48.6
		(20.1)	(22.8)	(26.5)	(24.3)
	Post	66.5	47.8	62	59.8
		(28)	(39.6)	(26.7)	(31.1)
I have felt aware of my emotions	Pre	73.3	72.8	75.8	74.1
		(20)	(17.7)	(14.9)	(17.2)
	Post	75.6	88.7	77.5	79.8
		(17.1)	(10.6)	(16.4)	(15.9)
My digestion has been good	Pre	66.6	67	65.4	66.3
		(34.1)*	(24.1)	(23.8)	(27.2)
	Post	71.2	72.9	71.3	71.7
		(18.6)	(28.5)	(22.1)	(22.1)
I have experienced self-doubt and insecurities	Pre	60	58.3	38.8	51.8
		(33.6)	(26.2)	(26.6)	(30.1)
	Post	48.4	50.7	34.6	44
		(32)	(36.1)	(21.8)	(29.9)
I have had good circulation	Pre	48.8	45.6	55.8	50.5
		(33.4)	(34.7)	(28)	(31.2)
	Post	68.8	60	63.8	64.6
		(23.2)	(26)	(30.3)	(26.1)
I have had a positive outlook on life	Pre	64.1	72.2	68.3	67.8
		(21)	(23.1)	(16.3)	(19.7)
	Post	75.8	67.1	78.9	74.6
		(14.4)	(29.8)	(17.6)	(20.6)
My head has felt clear and not foggy	Pre	51.3	58.3	58.8	55.9
		(25)	(25.7)	(17.3)	(22.3)
	Post	65.1	70.4	66.6	67.1
		(21.1)	(17.6)	(16.1)	(18)
I have felt grounded	Pre	47.5	52.2	53.8	51.1
		(21.7)	(29)	(16.9)	(21.9)
	Post	55.2	67.1	71.7	64.4
		(27.7)	(23.4)	(17)	(23.5)
I have found it easy to cope with life's stresses	Pre	51.3	62.6	56.3	56.2
		(21.8)	(22.9)	(19.8)	(21.2)
	Post	65.8	56.1	67.4	63.7
		(22.9)	(28.5)	(21.3)	(23.7)
I have felt aware of physical sensations in my body	Pre	73.7	68.7	72.1	71.7
		(13.6)	(16.3)	(27.7)	(20)
	Post	68.8	81.9	76.6	75.2
		(23.2)	(17)	(17.9)	(19.9)
I have felt in control of my emotions	Pre	59.6	62.1	65.8	62.5
		(23.4)	(20.4)	(17.7)	(20.2)
	Post	74.2	60.6	68.2	68.3
		(12.4)	(29.3)	(16.7)	(19.8)
I have enjoyed good interpersonal relationships	Pre	65.8	76.7	70	70.3
		(24.3)	(13.2)	(14.9)	(18.5)
	Post	48.4	50.7	34.6	44
		(32)	(36.1)	(21.8)	(29.9)

*Standard deviations are shown in parentheses.

## Appendices

See Tables 7 and 8.
